# Development of a Rapid and Efficient Method for Non-Lethal DNA Sampling and Genotyping in Scallops

**DOI:** 10.1371/journal.pone.0068096

**Published:** 2013-07-09

**Authors:** Junxia Mao, Jia Lv, Yan Miao, Changsen Sun, Liping Hu, Ru Zhang, Xiaoteng Fu, Lingling Zhang, Xiaoli Hu, Shi Wang, Zhenmin Bao

**Affiliations:** 1 Key Laboratory of Marine Genetics and Breeding, College of Marine Life Sciences, Ocean University of China, Qingdao, China; 2 School of Life Science, Taizhou University, Taizhou, China; University of Verona, Italy

## Abstract

Non-lethal DNA sampling has long appealed to researchers studying population and conservation genetics, as it does not necessitate removing individuals permanently from their natural environment or destroying valuable samples. However, such an approach has not yet been well established in bivalves. In this study, we demonstrate that the gill represents a good source of tissue for non-lethal sampling in scallops. Removal of a few gill filaments caused no noticeable behavioral abnormalities or increased mortality rates in Zhikong scallop (*Chlamys farreri*) during a three-month period of observation. To facilitate rapid gill-based DNA extraction, six methods (MA-MF) were designed and evaluated, each requiring less than one hour of processing time. The optimal method was identified as MF, in terms of maintaining DNA integrity and genotyping accuracy. Further optimization of MF method by orthogonal experimental design suggested that the utilization of gills could be limited to 2 mg of sample, which is sufficient for performing up to 20,000 PCR reactions. We also demonstrate the excellent cross-species utility of MF in two additional scallop species, Yesso scallop (*Patinopecten yessoensis*) and bay scallop (*Argopecten irradians*). Taken together, our study provides a rapid and efficient approach for applying non-lethal DNA sampling in bivalve species, which would serve as a valuable tool for maintaining bivalve populations and conservation genetics, as well as in breeding studies.

## Introduction

Scallops are a diverse group of animals consisting of more than 300 extant species that are distributed in ocean regions worldwide. It is well-established that they play a prominent role in the structure and function of local benthic ecosystems [1]. Many of these species are also of economic importance and support both commercial fisheries and mariculture efforts. With recent advances in molecular genetics, significant research attention has been provided to this particular group of animals for addressing scientific questions that are of ecological, evolutionary or economic importance. In genetic analyses, obtaining high-quality DNA samples represents a crucial step towards reliable data collection and statistical inference. However, the isolation of high-quality DNA is traditionally not a trivial task in molluscs due to the secretion of mucopolysaccharide and polyphenolic proteins that could copurify with DNA and interfere with the enzymatic processing of nucleic acids [2]. To address this issue, several specialized DNA extraction protocols [2]–[4], as well as commercial kits (e.g., E.Z.N.A.® Mollusc DNA kit from Omega Bio-Tek), have been developed that present modifications from those developed in mammals or plants. Unfortunately, these methods are generally laborious and time-consuming, and sometimes require a relatively large amount of sample materials that would have to sacrifice the animals under study to ensure sufficient amount of DNA for downstream applications.

Ideally, a non-lethal DNA sampling method, if convenient and cost-effective, would appeal to scallop researchers, as it would no longer necessitate removal of individuals permanently from the natural environment or destruction of valuable samples (e.g., endangered species). In certain circumstances, obtaining genetic information would only be useful if the animals under investigation remain alive. For example, in mark-recapture experiments, animals that receive a genetic tag prior to being released back into their natural habitat are recaptured to assess the survival and recruitment of a population [5]. In another instance, the marker-assisted selection (MAS) program screens a large number of individuals for desired genotypes, and selected individuals must remain alive to fulfill a pre-arranged mating design. Thus, sacrificing the animals to process their DNA would inhibit these applications. In general, these applications require a large number of samples, which calls for the development of an efficient non-lethal DNA sampling method that can be applied to the processing of a high number of samples.

For bivalves, non-lethal DNA sampling methods have been intensively investigated in mussels. In particular, the mantle-clipping method has been widely utilized for collecting tissue samples for genetic analyses [6]–[8]. However, this method was challenged in a recent study that described a high mortality rate and the appearance of shell deformity in the mantle-clipped snuffbox, *Epioblasma triquetra* [9]. To date, there is no report on developing a non-lethal DNA sampling method in scallops. This type of study should address two important issues: (i) determination of an appropriate source of tissue for sampling, with low or negligible effect on scallop viability; and (ii) development of an efficient DNA extraction protocol that can not only produce high-yield DNA of sufficient purity but can also facilitate rapid processing of a large number of samples.

In this study, to establish a non-lethal DNA sampling method in scallops, we initially investigated whether the gill could serve as a good tissue source in Zhikong scallops (*Chlamys farreri*). Next, six rapid DNA extraction methods were devised and evaluated in three key aspects (i.e., DNA integrity, long-term storage and genotyping accuracy), and the best method was further optimized by orthogonal array testing to minimize gill usage, while maintaining a high accuracy of genotyping. The cross-species utility of the best method was also evaluated in two scallop species, *Patinopecten yessoensis* (Yesso scallop) and *Argopecten irradians* (bay scallop).

## Materials and Methods

### Ethics Statement

Scallop handling was conducted in accordance with the guidelines and regulations that have been established by the Ocean University of China and by the local government.

### Scallop Materials

Adult Zhikong scallops (*C. farreri*) and bay scallops (*A. irradians*) were obtained from the Xunshan aquatic hatchery (Shandong Province, China), and Yesso scallops (*P. yessoensis*) were obtained from the Zhangzidao aquatic hatchery (Dalian Province, China).

### Gill Filament Sampling and Survival Rate Scoring

**Figure 1:**
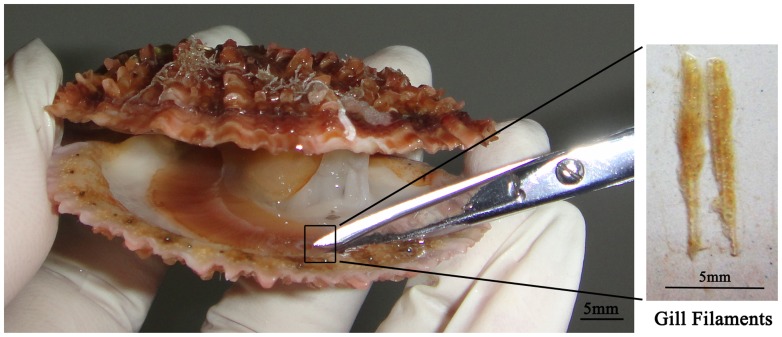
Gill filament sampling.

To minimize the sampling effects on the scallops, our gill sampling method consisted of gently forcing the shell open to a gape of ∼10 mm and then clipping 2–3 gill filaments (approximately 5 mg) from each individual at the anterior position, which was two-thirds of the total filament length, using a fine scissor (Fig. 1). These gill filaments were either used immediately or preserved at −20°C until further use. After gill sampling, the scallops were released back into water tanks where the same number of individuals that were not handled were also being held. Three replicates were arranged for each group, and each replicate contained 30 individuals. Seawater changes and feeding were performed twice daily, and the seawater temperature was kept at 17°C. Approximately three months after gill sampling, the survival rates were recorded for both groups (i.e., sampled vs. not sampled).

### Comparison of DNA Yield between Gill and Mantle

To evaluate whether the gill could serve as a good substitute for the mantle, DNA yield between gill and mantle tissue was compared. For the mantle sampling, we followed the method described in [10]. Genomic DNA was extracted from the same amount of gill and mantle materials, using the phenol-chloroform method [11]. The yield of DNA was compared among three different tissue amounts (0.10 g, 0.15 g and 0.20 g), using three Zhikong scallops. The DNA concentration was measured using the NanoVue plus spectrophotometer (GE Healthcare, UK).

### Design of Six Methods for Rapid DNA Extraction in Gills

To enable rapid isolation of genomic DNA from several gill filaments, six DNA extraction methods were devised and evaluated. The technical details of the six methods are described below and are briefly summarized in Table 1.

**Table 1:** Overview of the six rapid gill-based DNA extraction methods.

Main reagents and steps	MA	MB	MC	MD	ME	MF
Chelex-100 resin	√		√		√	
Proteinase K			√	√	√	√
SDS					√	√
56°C incubation			√	√	√	√
Boiling	√	√	√	√	√	√
Time consumed (min)	∼10	∼10	∼40	∼40	∼40	∼40
Genotyping accuracy (%)^a^	46.7±0.12	26.7±0.12	100±0.00	100±0.00	100±0.00	100±0.00

a values represent: mean±standard deviation.

- *Method A (MA) - Chelex-100 boiling.* The gill filaments were suspended in 200 µL of a 5% Chelex-100 resin solution (Bio-Rad, USA), and then the solution was boiled for 10 min to release genomic DNA from the cells. After cooling on ice, the cell lysis solution was centrifuged at 12,000 rpm for 10 min, and then the supernatant was transferred to a new tube and stored at −20°C until further use.
- *Method B (MB) - Water boiling.* This method was similar to MA; however, the Chelex-100 resin solution was replaced with sterile distilled water.
- *Method C (MC) - Chelex-100 *&* PRK digestion.* The gill filaments were suspended in 200 µL of a digestion solution containing 100 mM NaCl, 10 mM Tris-HCl, 1 mM EDTA (pH 8.0), 0.3 mg/mL Proteinase K (Roche, Switzerland) and 5% Chelex-100 resin (Bio-Rad, USA). The digestion solution was incubated at 56°C for 30 min and, then, boiled for 10 min to inactivate Proteinase K. After cooling on ice, the digestion solution was centrifuged at 12,000 rpm for 10 min, and the supernatant was transferred to a new tube and stored at −20°C until further use.
- *Method D (MD) - PRK digestion.* This method was similar to MC; however, Chelex-100 resin was not present in the digestion solution.
- *Method E (ME) - Chelex-100, SDS *&* PRK digestion.* This method was similar to MC; however, sodium dodecyl sulfate (SDS; Sigma, USA) was present in the digestion solution at a final concentration of 0.5%.
- *Method F (MF) - SDS *&* PRK digestion.* This method was similar to ME; however, Chelex-100 resin was not present in the digestion solution.

### Agarose Gel Electrophoresis Analysis of DNA Integrity

To determine the integrity of the DNA samples prepared by the six methods, 3 µL of each DNA sample was separated on a 1% agarose gel at 100 V for 25 min and was then visualized under UV light after staining with ethidium bromide.

For evaluation of long-term DNA storage, the same DNA samples were repeatedly subjected to agarose gel electrophoresis analysis after one month, five months and twelve months of storage.

### Single Nucleotide Polymorphism (SNP) Genotyping Assays

To evaluate the utility of the DNA samples prepared by the six methods for genotyping purposes, high-resolution melting (HRM) assays were set up for five SNP loci (Table 2) that were identified in the transcriptome of Zhikong scallop [12]. PCR amplification was performed in a 10 µL volume composed of 1 µL of a dilute DNA sample (1/100) or 20 ng of phenol-chloroform extracted DNA, 0.1 µM forward primer, 0.5 µM reverse primer, 1.5 mM MgCl_2_, 200 µM dNTP, 1 x LC Green Plus (Roche, Switzerland), 1 x PCR Buffer and 0.5 U of *Taq* polymerase (Takara, China). The PCR programs began with an initial denaturation step at 95°C for 5 min, followed by 60 cycles of 95°C for 40 s, 63°C for 40 s and 72°C for 40 s, and then a final extension step was performed at 72°C for 5 min. After PCR amplification, an aliquot of the appropriate probe was added to each reaction to a final concentration of 5 µM, and a mixture of the PCR product and probe was denatured at 95°C for 10 min, followed by an annealing step at 25°C for 1 min. HRM analysis was performed on the Light Scanner instrument (Idaho Technology, USA) with continuous signal acquisition during a 0.1°C/s ramp from 40°C to 95°C. The HRM data were retrieved and analyzed using LightScanner Call-IT 2.0 software.

**Table 2:** PCR primers and probe sequences of five *C. farreri* SNP markers.

Locus name	SNP type	Forward primer (5′→3′)	Reverse primer (5′→3′)	Probe sequences (5′→3′)
C591S171	T/C	TACGGACAGAACAGGTCACG	GGCCAGCTGACCTTGACTAC	CCCCTACTAGGTA[T]CAGACAGAATGGTCTG
C7293S162	C/A	GATTTTGTGGGAGCAAAGGA	CAGGGTTTGATTCATCCAGAA	TATGAAAACACAAG[C]AGTCATCCTTAATGGAT
C11303S437	C/T	AGGCGGACCTAAACACTTCA	TGTCCTCCTTTCCGCTTATG	TGTGGAACTATCCC[C]TCATACAAAGACCTTA
C3737S763	G/A	ACTCCGTAACCAACGACCTG	CACGCCTCCTTCCAGATGTA	CTGCAATTCTCTTC[G]AAATAGATACCACTAG
C5682S266	A/G	CGCTTGATTCCTTGACCTGT	AGAAACCTGTCCACACAATGG	ATCAAATATTTCGCT[G]ACCAATGGTGTTACACA

Note that for each probe, the SNP position was indicated by brackets.

### Optimization of MF using Orthogonal Array Testing

The orthogonal experimental design strategy was adopted to further optimize MF, to minimize the amount of gill usage, while maintaining a high genotyping accuracy. Three key factors (i.e., gill usage, SDS concentration and DNA dilution ratio) that possibly affect genotyping accuracy were included in the orthogonal experimental design, and four levels of values were evaluated for each factor (Table 3). Orthogonal arrays were obtained using the SPSS Statistics 17.0 software (Table 4). For each orthogonal array, HRM genotyping was performed using five SNP loci (Table 2) and three Zhikong scallops. DNA samples that were extracted using the phenol-chloroform method were also subjected to HRM genotyping to serve as controls. The range and variance analyses were performed according to a previously described method [13] to obtain the optimal factor configuration and to identify the contribution of each factor to the genotyping accuracy.

**Table 3:** The factors and corresponding levels used for orthogonal experimental design.

	Gill mass (mg) (A)	SDS concentration (g/ml) (B)	Dilution ratio (C)
1	2.0	0.25%	1∶10
2	5.0	0.50%	1∶10^2^
3	10.0	0.75%	1∶10^3^
4	20.0	1.00%	1∶10^4^

**Table 4:** L_16_ (4^3^) orthogonal arrays and their corresponding genotyping accuracy.

Exp. No.	Gill mass (mg) (A)	SDS concentration (g/ml) (B)	Dilution ratio (C)	Genotyping accuracy (%)^a^
1	2.0	0.25%	1∶10	6.7±0.12
2	5.0	0.75%	1∶10	6.7±0.12
3	10.0	1.00%	1∶10	0.0±0.00
4	20.0	0.50%	1∶10	0.0±0.00
5	2.0	0.50%	1∶10^2^	100.0±0.00
6	5.0	1.00%	1∶10^2^	93.3±0.12
7	10.0	0.75%	1∶10^2^	93.3±0.12
8	20.0	0.25%	1∶10^2^	100.0±0.00
9	2.0	0.75%	1∶10^3^	80.0±0.20
10	5.0	0.25%	1∶10^3^	93.3±0.12
11	10.0	0.50%	1∶10^3^	93.3±0.12
12	20.0	1.00%	1∶10^3^	100.0±0.00
13	2.0	1.00%	1∶10^4^	26.7±0.12
14	5.0	0.50%	1∶10^4^	26.7±0.12
15	10.0	0.25%	1∶10^4^	26.7±0.12
16	20.0	0.75%	1∶10^4^	40.0±0.35

a , values represent: mean±standard deviation.

Note, orthogonal array testing is an optimization method that enables the deduction of an optimal factor configuration using a minimum number of experiments rather than all the possible factor combinations (i.e. exhaustive testing). Here, only 16 orthogonal arrays that were selected from a total of 64 orthogonal arrays using the software SPSS Statistics 17.0 were subjected to experimental evaluation.

### Evaluation of the Cross-species Utility of MF

To investigate the cross-species utility of MF, the optimized MF method was applied to both Yesso and bay scallops. The DNA integrity was determined via agarose gel electrophoresis analysis. For genotyping assays, three simple sequence repeat (SSR) markers (FJ262381, FJ262399 and FJ262401) were obtained from a previous study [14] and utilized for Yesso scallop, and three markers (AIMS009, AIMS012 and AIMS022) obtained from previous studies [15], [16] were utilized for bay scallops. For each species, three individuals were utilized in SSR genotyping analyses, following the protocols described in the original references. DNA samples that were extracted using the phenol-chloroform method were also subjected to SSR genotyping to serve as controls.

## Results

### Non-lethal Gill Sampling

Our non-lethal gill sampling method required 2–3 gill filaments from each scallop (Fig. 1), which roughly provided 5 mg of gill material for downstream DNA preparation. The sampling effect was monitored for Zhikong scallops during a three-month period after gill sampling. No behavioral abnormalities were observed for the sampled groups. The average survival rate of the sampled groups was 86.7±3.35%, showing no significant difference from the unsampled groups (87.8±1.91%). These results suggest that our gill sampling method has a negligible impact on the viability of scallops.

### Comparison of DNA Yield between Gill and Mantle

We further evaluated whether the gill could serve as a good substitute for the mantle with respect to DNA yield. In this way, genomic DNA was extracted from the same amount of gills and mantles from three Zhikong scallops using a different amount of starting material. Interestingly, a comparison of the DNA yield revealed that the gill always produced approximately twice the amount of DNA than the mantle (Table 5), suggesting that, compared with the mantle, a smaller amount of gill material is needed to obtain the same amount of DNA.

**Table 5:** Comparison of DNA yields between mantle and gill.

Tissue weight (g)	N^a^	DNA yield of mantle (ug)^b^	DNA yield of gill (ug)^b^
0.10	3	10.17±1.26	21.34±0.47
0.15	3	13.18±0.36	26.83±1.02
0.20	3	23.66±2.46	48.63±6.18

a , number of individuals tested.

b , values represent: mean±standard deviation.

### Evaluation of Six Rapid DNA Extraction Methods by Agarose Gel Electrophoresis Analysis

**Figure 2:**
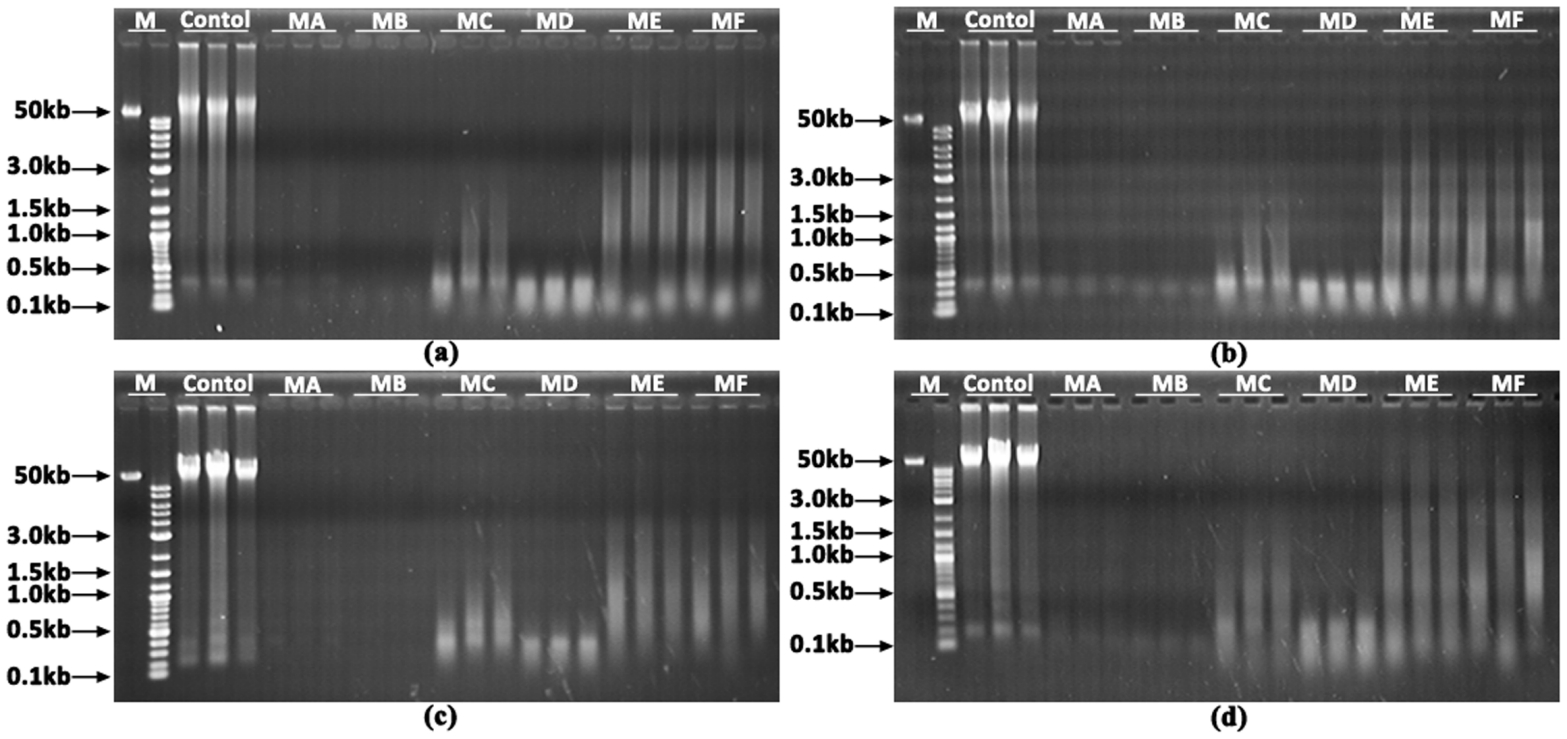
Agarose gel electrophoresis analysis of DNA samples from gills, prepared by six rapid DNA extraction methods (MA-MF) in Zhikong scallop. (a) Freshly prepared DNA samples; (b–d) DNA samples preserved for one month, five months and twelve months, respectively. M: DNA ladders; Control: DNA samples extracted by the phenol-chloroform method; MA-MF: DNA samples prepared by methods A–F.

To enable rapid isolation of genomic DNA from gill filaments, six DNA extraction methods (MA-MF) were devised and evaluated, comprising less than one hour of processing time. DNA samples prepared by the six methods were subjected to agarose gel electrophoresis analysis to determine the integrity of the DNA. In comparison with the traditional phenol-chloroform method, all of the six methods produced a smeared DNA pattern with differences in intensity and range (Fig. 2a). For MA and MB, cell lyses were usually incomplete. In accordance with this observation, the intensities of the DNA smears were much weaker with MA and MB than with the other methods. Compared with MB, MA produced a longer, though weak, DNA smear (0.1–3 kb), possibly due to the presence of Chelex-100 resin in the lysis solution. In comparison with MA and MB, MC and MD produced a similar DNA smear, but with much stronger intensity, suggesting that Proteinase K can improve the digestion efficiency. The longest and strongest DNA smears (0.1–50 kb) were observed with ME and MF, with no noticeable difference between them, suggesting that SDS can remarkably improve the digestion efficiency and protect the DNA integrity.

### Evaluation of Long-term DNA Storage

The effect of long-term DNA storage was also evaluated for the six methods. Agarose gel electrophoresis analysis of DNA samples preserved for one month, five months and twelve months were shown in Fig. 2b–d. Even after twelve months of storage, DNA degradation was barely noticeable with the six methods, suggesting that DNA samples prepared by these methods can be well-preserved for at least twelve months.

### Evaluation of Six Rapid DNA Extraction Methods using SNP Genotyping Assays

**Figure 3:**
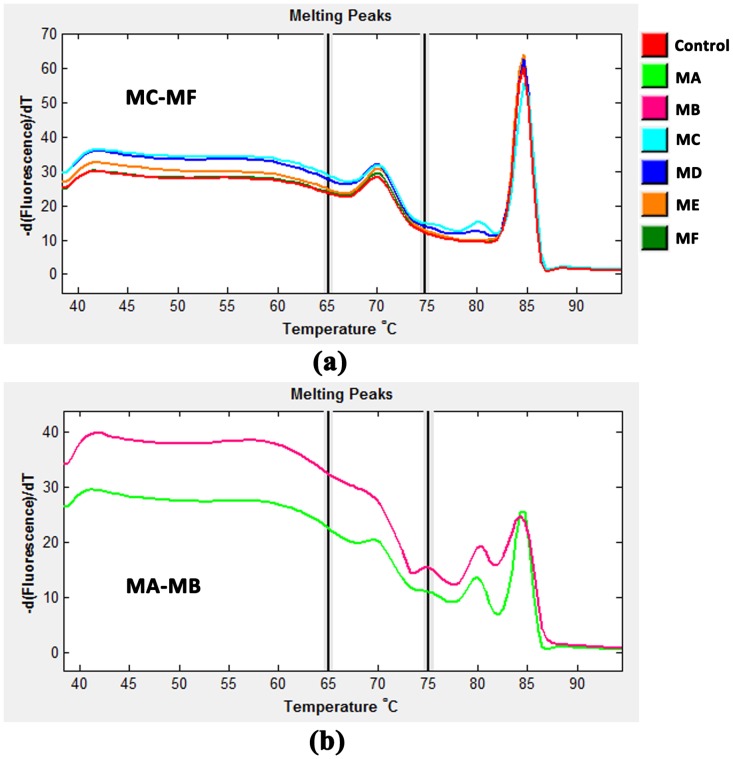
Normalized melting curves obtained by SNP genotyping (SNP locus - C591S171) of DNA samples prepared by the six methods from a single Zhikong scallop. (a) MC-MF and control; (b) MA and MB. Melting curve profiles were usually not clear for MA and MB.

We further evaluated the six methods for their suitability towards genotyping. HRM genotyping assays were designed for five SNP loci, using DNA samples that were prepared from three Zhikong scallops by the six methods. The genotyping accuracy of the six methods was shown in Table 1. The genotyping accuracy for both MA and MB was quite low (<50%), and the corresponding melting curves were usually not clear (Fig. 3b) compared with the other methods (Fig. 3a), possibly because of inefficient PCR amplifications due to extremely low concentrations of DNA. The genotyping accuracy of the other four methods all reached 100%, suggesting these methods can be reliably used in genotyping applications. Considering the DNA integrity, ME and MF outperformed the other methods due to their great potential for PCR amplification of much longer fragments. Because MF did not involve the relatively expensive reagent, Chelex-100 resin, MF was selected for further optimization.

### Optimization of MF by Orthogonal Array Testing

To minimize gill usage while maintaining high genotyping accuracy, MF was further optimized using the orthogonal experimental design strategy. Orthogonal arrays were designed for SNP genotyping with three key emphases: gill mass, SDS concentration and DNA dilution ratio (Table 4). The genotyping accuracy for each orthogonal array was shown in Table 4, which ranged from 0% to 100%. Genotyping accuracy was high (80–100%) for array numbers 5–12, and ≤40% for the others. In particular, when the dilution ratio was 1∶10 (array numbers 1–4), the genotyping accuracy was always below 10%.

The value range analysis revealed that dilution ratio had the greatest influence on the genotyping accuracy, followed by gill mass and the SDS concentration (Table 6). The best factor configuration was identified as A1B2C2 (i.e., 2 mg of gills, 0.5% SDS, and 1∶100 dilution ratio) when taking both gill usage and genotyping accuracy into account. The variance analysis also revealed that the dilution ratio was a critical factor affecting the genotyping accuracy (Table 7).

**Table 6:** Value range analysis of three factors of MF.

	Gill mass (A)	SDS concentration (B)	Dilution ratio (C)
K_1_	2.134	2.267	0.134
K_2_	2.200	2.200	3.866
K_3_	2.133	2.200	3.666
K_4_	2.400	2.200	1.201
R (K_max_ – K_min_)	0.267	0.067	3.732

**Table 7:** Variance analysis of three factors of MF.

Factors	SS	df	MS	F	P
Gill mass	0.012	3	0.004	0.781	0.546
SDS concentration	0.001	3	3.33E-4	0.055	0.981
Dilution ratio	2.547	3	0.849	166.713	3.63E-6
Error	0.031	6	0.005		

SS: sum of squares; df: degrees of freedom; MS: mean square.

### Evaluation of the Cross-species Utility of MF

**Figure 4:**
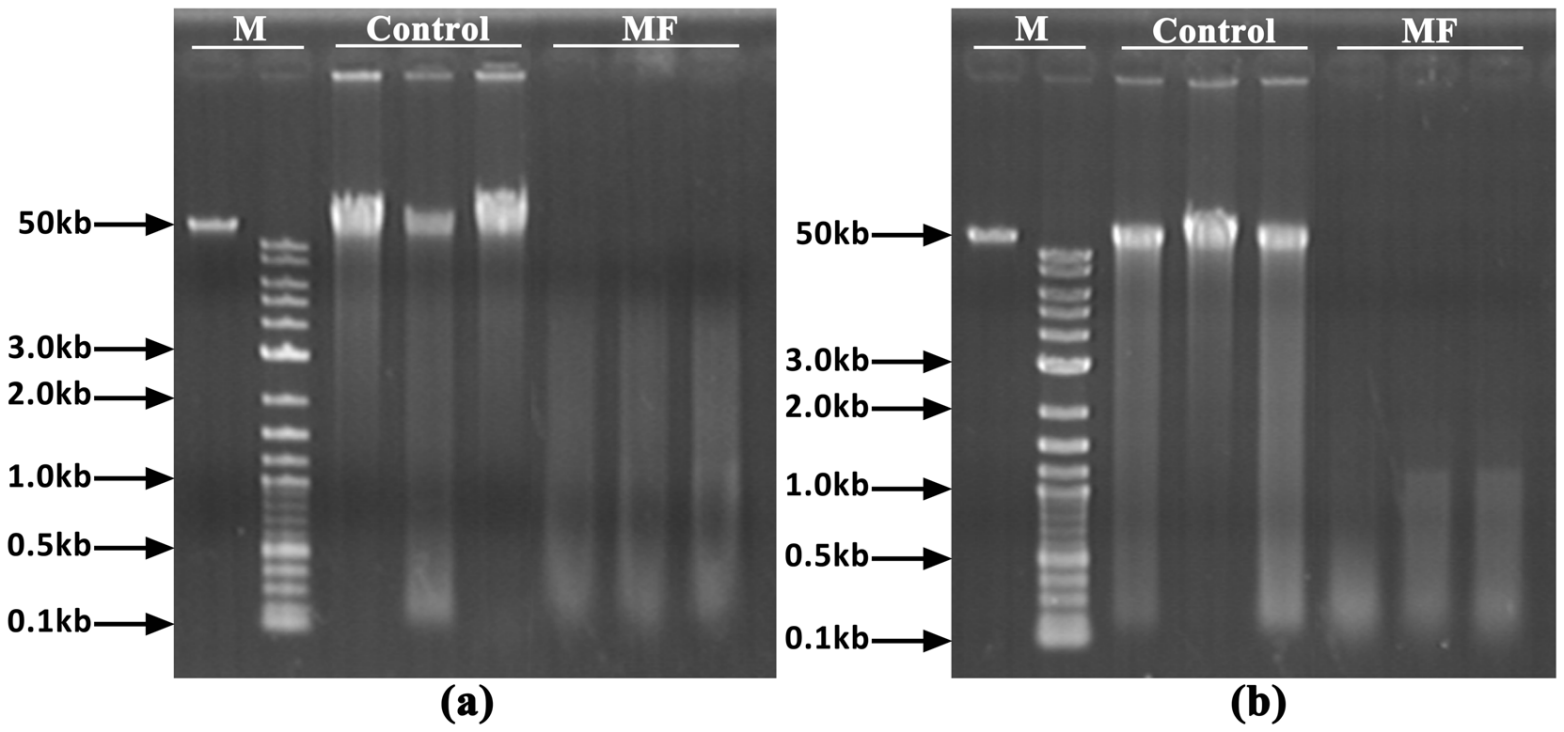
Agarose gel electrophoresis analysis of DNA samples prepared by MF in (a) Yesso scallop and (b) bay scallop. M: DNA ladders; Control: DNA samples extracted by the phenol-chloroform method.

**Figure 5:**
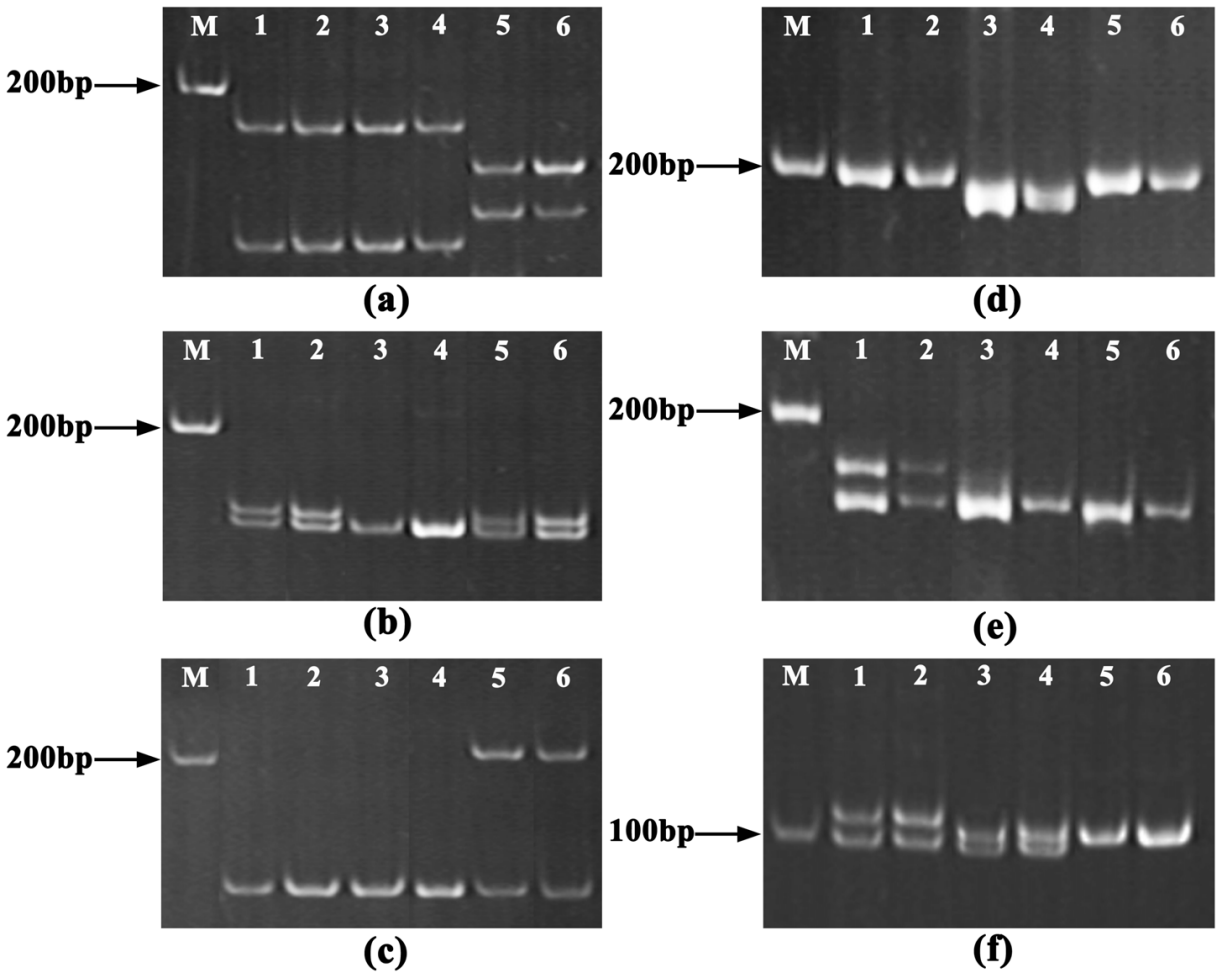
SSR genotyping of DNA samples prepared by MF in Yesso and bay scallops. (a–c) Genotyping results for SSR loci FJ262401, FJ262399 and FJ262381 in Yesso scallop; (d–f) Genotyping results for SSR loci AIMS022, AIMS012 and AIMS009 in bay scallop. Lanes 1, 3 and 5: DNA samples extracted by the phenol-chloroform method from three scallop individuals; lanes 2, 4 and 6: DNA samples prepared by MF from the same three individuals.

The optimized MF method was further applied to both Yesso and bay scallops, to evaluate its cross-species utility. Agarose gel electrophoresis analysis revealed a similar wide range of DNA smears (0.1–50 kb) in Yesso scallop (Fig. 4a), but a narrow range of DNA smears (0.1–2 kb) in bay scallop (Fig. 4b), indicating a difference in DNA integrity obtained by this method in the two scallop species. The genotyping results were shown in Fig. 5a–c for Yesso scallop and Fig. 5d–f for bay scallop. Thus, all SSR loci could be successfully amplified, and the genotyping accuracy all reached 100% in the two scallop species.

## Discussion

### Tissue Selection for Non-lethal DNA Sampling in Scallops

The development of an effective non-lethal DNA sampling method would require that the source tissue be carefully determined, with minimal effects on the animals. The mantle-clipping method is a commonly used non-lethal DNA sampling method in bivalve species (e.g., mussels and oysters); however, this method has recently been challenged due to the observation of a high mortality rate and shell deformity [9]. Our study demonstrates that the gill can serve as a good substitute for the mantle for non-lethal DNA sampling in scallops. One advantage of the use of the gill for non-lethal DNA sampling is that the scallop gill can produce almost twice the amount of DNA than the mantle. It suggests that in comparison with the mantle, less amount of the gill is needed to produce the same amount of DNA, which, in turn, can reduce the potential sampling effects on scallops. In addition, we have shown that removal of a few gill filaments would not cause noticeable behavioral abnormalities or increased mortality rates during a three-month period of observation. Taken together, we conclude that the scallop gill represents the appropriate source tissue for non-lethal DNA sampling in scallops.

### Development of an Optimal Method for Rapid Gill DNA Extraction

Isolation of genomic DNA from only a few gill filaments requires the development of an efficient DNA extraction method. To achieve this goal, six methods were designed by selective combination of several key reagents (i.e., Chelex-100 resin, Proteinase K, and SDS) to assess their impacts on cell lysis efficiency and genotyping accuracy. All six methods required less than one hour of processing time, and the resultant DNA samples can be safely preserved for at least twelve months without noticeable degradation. It has been shown that Chelex-100 resin can prevent DNA degradation by chelating metal ions that may otherwise catalyze the breakdown of DNA subjected to high temperatures in low ionic strength solutions [17], [18]. Consistent with previous studies, our study has clearly shown that the presence of Chelex-100 resin in the lysis solution could increase the molecular weight of DNA samples (e.g., compare MA vs. MB and MC vs. MD). However, when it worked alone (i.e., MA), the concentration of the DNA samples remained quite low, resulting in low genotyping accuracy (<50%). Addition of Proteinase K can alleviate this problem by improving the digestion efficiency, but the size range of the obtained DNA samples was not very long (0.1–3 kb). Although this size range does meet the basic requirements of most genotyping studies, it has limitations for the amplification of much longer fragments. Most importantly, we demonstrate that the presence of SDS in the lysis solution could remarkably improve cell lysis efficiency and could produce high molecular weight DNA fragments (0.1–50 kb). There may be concerns regarding the use of such DNA samples for PCR amplification, as SDS can potentially inhibit the activity of *Taq* polymerase [19]. However, this issue can be resolved when the final SDS concentration in a PCR reaction is controlled at a low level. As demonstrated in this study, 100% genotyping accuracy could be achieved with ME and MF, where the final SDS concentration in a PCR reaction was extremely low (0.0005%). Comparison of MF with ME revealed no noticeable difference in cell lysis efficiency, as well as genotyping accuracy, suggesting that Chelex-100 resin is not necessary when SDS is present in the lysis solution. As MF does not require this relatively expensive reagent, Chelex-100 resin, the MF method is considered to be optimal for non-lethal DNA sampling in scallops.

### Orthogonal Experimental Design for MF Method Optimization

Orthogonal experimental design is an optimization method for researching multiple factors and levels [13]. It enables the deduction of an optimal factor configuration using a minimum number of experiments. Although powerful, it is seldom used for optimization of DNA extraction protocols. In the present study, this strategy was adopted for further optimization of MF to minimize gill usage, while maintaining high genotyping accuracy. When taking into account both gill usage and genotyping accuracy, the best factor configuration was identified as A1B2C2, in which only 2 mg of gill material is required to produce a sufficient amount of DNA for up to 20,000 PCR reactions.

Orthogonal array testing also enables the statistical analysis of the effectiveness of each factor. Among the three factors (i.e., gill mass, SDS concentration, and dilution ratio) that were evaluated for MF, analyses of the value range and the variance revealed that the dilution ratio had the greatest influence on the genotyping accuracy, possibly due to its major role in regulating the SDS and DNA concentrations in the PCR reaction. A significantly low dilution ratio (e.g., 1∶10) would increase the SDS concentration to a point that would inhibit the PCR reaction, while a high dilution ratio (e.g., 1∶10^4^) could lead to PCR failure due to the lack of a sufficient amount of DNA template for initiating PCR amplification.

### The Cross-species Utility of MF

Because the composition of the cellular components that can either break down DNA or interfere with the PCR reactions may be quite different in various scallop species, a non-lethal DNA extraction method developed in one species may not necessarily be applicable to other species. In this study, the cross-species utility of MF was evaluated in two scallop species, Yesso and bay scallops. We demonstrated that the optimized MF method could be efficiently applied to Yesso and bay scallops, and the genotyping accuracy reached 100% in both species. Notably, we observed discrepancies in agarose gel electrophoresis assays of both species. The size ranges of the DNA smears from bay scallops were much shorter (0.1–2 kb) than those of Zhikong and Yesso scallops. Such discrepancies may be resolved in future studies of species-specific optimization for MF method.

In conclusion, we established a rapid and efficient method for non-lethal DNA sampling in scallops, which has been thoroughly evaluated and proven to be utilized in genotyping applications, without sacrificing the studied animals. Our study provides a valuable tool for facilitating population and conservation genetics, as well as breeding studies, in scallops.
